# Letter to the editor in response to ‘Reconstruction and prediction of viral disease epidemics’

**DOI:** 10.1017/S095026881900013X

**Published:** 2019-02-22

**Authors:** T. Alex Perkins

**Affiliations:** Department of Biological Sciences and Eck Institute for Global Health, University of Notre Dame, 100 Galvin Hall, Notre Dame, IN 46556, USA

## To the Editor

The article by Kraemer *et al*. [1], ‘Reconstruction and prediction of viral disease epidemics,’ provides a nice review of several tools used in the analysis of viral disease epidemics and calls for increased, and timelier, integration of these tools. One interesting feature of this paper is the presentation of publication dates for key papers alongside incidence time series for each of four epidemics. I am writing because this presentation neglects a crucial consideration about the publication of scientific research during disease epidemics: the use of preprints.

The importance of preprints and other forms of information sharing in the context of disease epidemics has been formally recognised by numerous funders and publishers of scientific research [2]. Of the 16 papers highlighted by Kraemer *et al*., only five published their findings as preprints in advance of final publication in a peer-reviewed journal (Table 1). Doing so accelerated the availability of those findings by 111–222 days. Three others published their findings in *PLOS Currents Outbreaks*, a now defunct outlet that involved peer review but was otherwise known for its expeditious publication of research during disease epidemics. The other eight withheld their findings from the public domain during critical times when that information could have proven useful for public health officials and other researchers. It is worth noting though that as the use of preprints during disease epidemics grows, so too do challenges associated with their use and the need for solutions to enhance their adoption and impact [3].

**Table 1.** Dates of peer-reviewed publication and, where applicable, preprint publication for 16 studies highlighted by Kraemer *et al*. [1]

	Preprint publication date	Peer-reviewed publication date
Zika virus outbreak in the Americas (2014–2017)		
Geographic origin	NA	24 March 2016
Geographic spread	NA	14 January 2016
Number of cases	12 February 2016 doi:10.1101/039610	25 July 2016
Integration of multiple data types	2 February 2017 doi:10.1101/105171	24 May 2017^a^
Integration of multiple data types	3 February 2017 doi:10.1101/104794	24 May 2017^a^
Ebola virus disease outbreak in West Africa (2013–2016)		
Geographic origin	NA	12 September 2014^a^
Geographic spread	NA^b^	2 September 2014
Number of cases	NA^b^	8 September 2014^a^
Integration of multiple data types	2 September 2016 doi:10.1101/071779	12 April 2017
Yellow fever outbreak in Central Africa (2015–2016)		
Geographic origin	NA	20 September 2016
Geographic spread	NA	22 December 2016
Number of cases	NA	16 January 2018
Integration of multiple data types	NA	NA
Middle East Respiratory Syndrome (MERS) outbreak (2012–2017)		
Geographic origin	NA	20 September 2013
Geographic spread	NA^b^	17 July 2013
Number of cases	NA	5 July 2013
Integration of multiple data types	10 August 2017 doi:10.1101/173211	16 January 2018

For each peer-reviewed publication, a corresponding preprint publication was searched for on four prominent preprint servers: bioRxiv, arXiv, F1000Research and PeerJ Preprints. A digital object identifier (doi) is provided for each preprint identified.

a Corrected from dates reported in Table 1 by Kraemer *et al*.

b Published in *PLOS Currents Outbreaks*.

In neglecting the issue of preprints, Kraemer *et al*. also missed some interesting observations. Consideration of preprint dates makes clearer that three types of findings (geographic origin, geographic spread, number of cases) were all reported relatively close to one another during fairly early stages of three of the epidemics (Zika, Ebola, MERS). In the other epidemic (yellow fever), all three peer-reviewed publications lagged well behind the epidemic, although two were published soon enough that preprint sharing could have potentially made their findings available while the epidemic was still ongoing. Publication of integrated analyses lagged behind all three of the other analysis types by no less than 10 months. This dichotomy illustrates that the integrated analyses advocated by Kraemer *et al*. are the ones that struggle the most with timely publication, although all four integrated analyses did make preprints available months in advance of their peer-reviewed form.

In closing, it is worth reflecting on the rather ironic possibility that this letter may not have been necessary had Kraemer *et al*. released an early draft of their work as a preprint. Doing so would have allowed for a wider range of input from those not involved in the process of formal peer review at *Epidemiology and Infection*. Similar value applies to the use of preprints to disseminate time-sensitive findings during disease epidemics.
